# Effectiveness of a Hospital-Based Computerized Decision Support System on Clinician Recommendations and Patient Outcomes: A Randomized Clinical Trial

**DOI:** 10.1001/jamanetworkopen.2019.17094

**Published:** 2019-12-11

**Authors:** Lorenzo Moja, Hernan Polo Friz, Matteo Capobussi, Koren Kwag, Rita Banzi, Francesca Ruggiero, Marien González-Lorenzo, Elisa G. Liberati, Massimo Mangia, Peter Nyberg, Ilkka Kunnamo, Claudio Cimminiello, Giuseppe Vighi, Jeremy M. Grimshaw, Giovanni Delgrossi, Stefanos Bonovas

**Affiliations:** 1 Department of Biomedical Sciences for Health, University of Milan, Milan, Italy; 2 Clinical Epidemiology Unit, Istituto di Ricovero e Cura a Carattere Scientifico (IRCCS) Orthopedic Institute Galeazzi, Milan, Italy; 3 Internal Medicine Division, Medical Department, Vimercate Hospital, Vimercate, Italy; 4 Medical School of International Health, Ben Gurion University of the Negev, Beer Sheva, Israel; 5 IRCCS Mario Negri Institute for Pharmacological Research, Milan, Italy; 6 Humanitas Clinical and Research Center, Milan, Italy; 7 Department of Biomedical Sciences, Humanitas University, Milan, Italy; 8 The Healthcare Improvement Studies Institute, University of Cambridge, Cambridge, United Kingdom; 9 Medilogy Srl, Milan, Italy; 10 Duodecim Medical Publications Ltd, Helsinki, Finland; 11 Clinical Epidemiology Program, Ottawa Hospital Research Institute and the Department of Medicine, University of Ottawa, Ottawa, Ontario, Canada

## Abstract

**Importance:**

Sophisticated evidence-based information resources can filter medical evidence from the literature, integrate it into electronic health records, and generate recommendations tailored to individual patients.

**Objective:**

To assess the effectiveness of a computerized clinical decision support system (CDSS) that preappraises evidence and provides health professionals with actionable, patient-specific recommendations at the point of care.

**Design, Setting, and Participants:**

Open-label, parallel-group, randomized clinical trial among internal medicine wards of a large Italian general hospital. All analyses in this randomized clinical trial followed the intent-to-treat principle. Between November 1, 2015, and December 31, 2016, patients were randomly assigned to the intervention group, in which CDSS-generated reminders were displayed to physicians, or to the control group, in which reminders were generated but not shown. Data were analyzed between February 1 and July 31, 2018.

**Interventions:**

Evidence-Based Medicine Electronic Decision Support (EBMEDS), a commercial CDSS covering a wide array of health conditions across specialties, was integrated into the hospital electronic health records to generate patient-specific recommendations.

**Main Outcomes and Measures:**

The primary outcome was the resolution rate, the rate at which medical problems identified and alerted by the CDSS were addressed by a change in practice. Secondary outcomes included the length of hospital stay and in-hospital all-cause mortality.

**Results:**

In this randomized clinical trial, 20 563 patients were admitted to the hospital. Of these, 6480 (31.5%) were admitted to the internal medicine wards (study population) and randomized (3242 to CDSS and 3238 to control). The mean (SD) age of patients was 70.5 (17.3) years, and 54.5% were men. In total, 28 394 reminders were generated throughout the course of the trial (median, 3 reminders per patient per hospital stay; interquartile range [IQR], 1-6). These messages led to a change in practice in approximately 4 of 100 patients. The resolution rate was 38.0% (95% CI, 37.2%-38.8%) in the intervention group and 33.7% (95% CI, 32.9%-34.4%) in the control group, corresponding to an odds ratio of 1.21 (95% CI, 1.11-1.32; *P* < .001). The length of hospital stay did not differ between the groups, with a median time of 8 days (IQR, 5-13 days) for the intervention group and a median time of 8 days (IQR, 5-14 days) for the control group (*P* = .36). In-hospital all-cause mortality also did not differ between groups (odds ratio, 0.95; 95% CI, 0.77-1.17; *P* = .59). Alert fatigue did not differ between early and late study periods.

**Conclusions and Relevance:**

An international commercial CDSS intervention marginally influenced routine practice in a general hospital, although the change did not statistically significantly affect patient outcomes.

**Trial Registration:**

ClinicalTrials.gov identifier: NCT02577198

## Key Points

**Question:**

Can a multispecialty computerized clinical decision support system (CDSS) reduce inappropriate prescribing in a general hospital?

**Findings:**

In this randomized clinical trial of 6480 patients, the CDSS alerted to a median of 3 reminders for prescription problems per patient per hospital stay. The CDSS led to a change in practice in approximately 4 of 100 patients, an effect that was maintained over time.

**Meaning:**

The CDSS was only marginally effective at reducing inappropriate medication prescribing compared with guidelines alone.

## Teaser

This randomized clinical trial assesses the effectiveness of a computerized decision support system that preappraises evidence and provides health professionals with actionable, patient-specific recommendations at the point of care.

## Introduction

The medical information market has evolved rapidly over the past decade, with innovative products (eg, point-of-care information summaries) gaining popularity among physicians. Accessibility to high-quality, well-summarized evidence-based information can help physicians identify the best therapeutic or diagnostic options for patients. Among the most successful products on the market are UpToDate, DynaMed, BMJ Best Practice, and EBM Guidelines.^1^ Recent developments in their ability to integrate into electronic health records (EHRs) allow these tools to act as clinical workflow–specific evidence-based information systems.^2^ The most advanced of such point-of-care information summary–generating systems are computerized clinical decision support systems (CDSSs), which are designed to link patient-specific information in EHRs with evidence-based knowledge to generate case-specific guidance messages through a rule-based or algorithm-based software program.^3,4^

The use of CDSSs has been proposed as a potential remedy for improving the overall efficiency and quality of health care.^5,6^ The CDSSs have been reported to encourage better adherence to evidence-based guidelines, improve the use of preventive measures, identify potential risks associated with the prescription of multiple medications, increase the availability of more accurate medical records, and enhance patient-physician communication.^7,8^ However, evidence on the efficacy of CDSSs for improving mortality or the course of diseases has been less consistent, with an unexplored potential for unintended consequences.^4^ Concerns may include the cost of maintaining the technology, greater bureaucratic demands, issues related to its usability (eg, irrelevant notifications and alert fatigue), clinicians’ fear of losing autonomy in decision-making, or a belief that the information technology may be used in medicolegal cases.^9^ The responsiveness of individual clinicians to the technology is difficult to predict and seems to depend on broader social and organizational factors, such as the quality of interdisciplinary relationships and an organizational ethos of transparency and accountability.^9^

The arrival of the latest generation of CDSSs on the market represents an opportunity for hospitals equipped with EHRs to adopt CDSSs, implementing literature surveillance systems and evidence appraisal performed by international publishing groups. These systems may better support clinical decision-making across multiple specialties and are accessible at competitive costs.^1^ The availability of CDSS content in multiple languages, improved format and usability of the reminders, and strategic marketing may draw point-of-care evidence beyond isolated research communities into mainstream use. However, acceptance of tools aimed at global use should rely on evidence of better care and improved patient outcomes. To our knowledge, these variables have never been empirically assessed in this setting.

The aim of the present study was to evaluate the effectiveness of a vendor-based multispecialty CDSS that generates patient-specific reminders based on high-quality point-of-care information services on clinical practice and quality of care in a general hospital (Vimercate Hospital, Vimercate, Italy). Reminders primarily targeted prescription medications.

## Methods

The Computerized Decision Support (CODES) trial was a pragmatic, open-label, parallel-group, randomized clinical trial (RCT) carried out between November 1, 2015, and December 31, 2016. Data were analyzed between February 1 and July 31, 2018. The RCT was conducted within the internal medicine wards of Vimercate Hospital, a large Italian nonteaching general hospital with an overall capacity of 489 beds that serves a population of approximately 200 000 inhabitants. Patients were admitted to the wards either from the emergency department of the hospital or from another hospital or were referred by primary care clinics. As a pragmatic RTC, the CODES trial enrolled all patients admitted into the internal medicine wards of the hospital during the course of the trial, without applying any exclusion criteria. The trial protocol is available in Supplement 1, and a version of the protocol has been published.^10^ The trial is reported in accordance with the 2001 Consolidated Standards of Reporting Trials (CONSORT) statement.^11^ Ethics approval for the conduct of this study was obtained from the Ethical Committee of the Monza and Brianza Province (Lombardy region, Italy), which granted a waiver of signed patient informed consent because the research involves only standard medical practices and no procedures for which written consent is normally required outside of the research context.

### Development of the CDSS

Vimercate Hospital electronically tracks all clinical and administrative information through a commercial EHR system (Tabula Clinica; Dedalus Italia SpA).^12^ We chose the CDSS after a comparative assessment of available commercial products on the market using a predefined set of essential criteria.^13,14^ Among the top-performing products, Evidence-Based Medicine Electronic Decision Support (EBMEDS) was selected because it offered the best conditions for system integration. Developed by Duodecim Medical Publications Ltd, an international publishing company owned by the Finnish Medical Society,^15^ EBMEDS includes algorithms from INXBASE (the Swedish Finnish Interaction X-referencing, formerly known as SFINX), a drug-drug interaction database developed by Medbase Ltd^16^ that contains concise evidence-based information about the harms and benefits of approximately 18 000 drug-drug interactions and adverse events,^17,18^ and from Renbase, a database of drug dosing recommendations in patients with renal failure.^19^ The CDSS was translated and adapted to the Italian health care setting by Medilogy Srl in collaboration with the University of Milan and was renamed Medilogy Decision Support System (MediDSS).^20^

### CDSS and EHR Integration

To generate patient-specific guidance and reminders, including therapeutic suggestions, EBMEDS integrates structured patient data from EHRs using the following controlled vocabularies and code sets: medical history, vital signs, diagnoses (*International Classification of Diseases*, *Ninth Revision*), symptoms (*International Classification of Primary Care*, *Second Edition*), medications (World Health Organization Anatomical Therapeutic Chemical Classification System), and immunization dates, allergies, and laboratory data (Logical Observation Identifiers Names and Codes), as well as imaging reports. Reminders were automatically generated and presented on screen when clinicians entered new information or accessed a patient’s EHR. We endeavored to integrate the CDSS into the clinical workflow by implementing alert features to grade different alerting priorities (eg, using color codes). However, we preferred noninterruptive reminders, which are locally more accepted, as opposed to interruptive alerts, which usually are associated with statistically significantly higher level of effectiveness.^21^ To control the security of the technology and to enable a smooth transition, the CDSS was sequentially integrated over 3 periods at different wards of the hospital. By the end of the trial, the intervention was active in all internal medicine wards of Vimercate Hospital.

### Patient-Specific Guidance

The present study used clinical support rules (n = 262) that cover a wide range of health conditions. Once the CDSS was integrated in a ward, all rules were implemented simultaneously. The format of the rules was broad, encompassing alerts and reminders, recommendations from clinical guidelines, condition-specific order sets, diagnostic support, and contextually relevant reference information.^22^ Guidance messages were targeted toward attending physicians and could be as simple as drug dose modification because of reduced renal clearance or as complex as medication recommendation based on scoring algorithms extracting data from test results and clinical assessments. Suggestions were based on international evidence–based point-of-care summaries, including Cochrane systematic reviews.^23,24^ Quality control of guidance was based on independent revision of contents by an international panel of experts led by Duodecim Medical Publications Ltd and secondary revision by clinicians from the trial site. Examples of specific patient guidance are reported in the Results section.

Because combinations of drugs can trigger alerts on thousands of drug-drug interactions and adverse events, alerts were organized and presented based on severity following the taxonomy adopted by the Medbase software program (Medbase Ltd).^16^ This database classifies drug-drug interactions into categories of clinical relevance (ranging from A for minor interaction to D for combination best avoided) and level of documentation (ranging from 0 for extrapolation from studies with similar drugs to 4 for availability of controlled studies), refining the earlier, widely accepted Swedish interaction classification system by Sjöqvist.^25^

Guidance on EBMEDS, including a reference source for potentially inappropriate care, was available to physicians seeing patients in the intervention group. Therapeutic-specific and diagnosis-specific links to full-text guidelines were available in the control group, representing modestly enhanced usual care.

### Randomization and Blinding

Patients were randomized through their anonymous patient identification numbers. The computer-generated allocation to each study arm on a 1:1 basis was stratified by sex and age using permuted blocks of random sizes. We randomized patients based on the assumption that the EHR operates primarily at the level of the individual patient. However, this choice created a potential for cross-contamination and dilution of the effect size. We reasoned a priori that it is unlikely that physicians might have the opportunity to discuss details of hundreds of reminders among themselves, limiting the risk of contamination.^26,27^ Once patients were randomized to a study arm, they remained in that arm for all subsequent hospital admissions. The project team, including hospital investigators and statisticians (H.P.F., C.C., G.V., and G.D.), was blinded to the allocation during the conduct of the trial. Physicians were not blinded.

### Outcomes

The primary outcome was the resolution rate, the rate at which medical problems identified and alerted by the CDSS were addressed or resolved by physicians through a change in practice. The CDSS tracked all clinical problems that triggered an alert regardless of the treating physician. Alerts were checked for quality and relevance at multiple levels (eg, through the EBMEDS Quality Plan,^28^ external expert review, feedback on user experience, and proactive monitoring of perceived effectiveness by users). We also considered the median time to resolution of the reminders, specifically the time between the activation of the warning message and its resolution.

Secondary outcomes explored resolution rates for different types of reminders (eg, EBMEDS clinical reminders, class C drug interactions, class D drug interactions, and reminders developed by the project team based on specific hospital needs), as well as clinical outcomes, including the following: (1) in-hospital all-cause mortality (measured primarily for safety reasons) and mortality at 30 days and 90 days (ie, mortality for any reason within 30 days and 90 days after hospital admission), (2) mortality related to venous thromboembolism (VTE), (3) in-hospital morbidity for VTE-related causes, and (4) the length of hospital stay (measured as a process outcome) during the study period. The outcomes prespecified in the trial registration are the same as those in the published protocol,^10^ all of which are reported in this article. However, we also added 2 more time points for mortality (at 30 days and 90 days), whereas our protocol prespecified only in-hospital mortality. Sample sizes were not calculated for secondary outcomes.

The EHRs were used as sources of clinical and nonclinical data for the purpose of this RCT. Because Vimercate Hospital has high patient retention, EHRs were thoroughly completed and coded, including patients’ medical history. Mortality data were obtained from the Lombardy region death registry. The research team had no control over the EHRs.

The RCT drew on qualitative analysis of data from interviews with clinicians (physicians and nurses) and hospital senior management carried out before and during the trial to assist in identifying the burden of the intervention and potential barriers (eg, irrelevant notifications and alert fatigue) to its implementation.^9^ A set of tailored training seminars to help facilitate the effective adoption of the technology was implemented in the first trial period. No concurrent patient safety or quality improvement initiatives were conducted at the time of the trial.

### Sample Size

We estimated the sample size based on the primary outcome (the resolution rate). We assumed baseline resolution rates of 5% in the intervention group vs 3% in the control group (ie, 2% absolute difference). This estimated difference was informed by findings from a systematic review on the association between computer reminders at the point of care and processes and outcomes of care, which reported a mean reduction of 4.2% from the baseline resolution rate.^29^ To account for potential contamination of knowledge between the intervention and control groups in our study, we halved the target from 4.2% to 2%. With 4230 guidance messages generated across both study arms, the study would have 90% power to detect a difference between the 2 groups (2-sided α = .05, 1:1 allocation). We increased the required sample size by 10% (decided arbitrarily) to 4650 reminders to account for clustering by patient. Based on a prior study^30^ evaluating EBMEDS, which recorded a mean of 0.30 reminders per individual triggered at baseline, we determined that 15 500 patients (7750 per group) needed to be enrolled in the study. The design used a sample size reestimation at the interim analysis after 50% of the expected events had occurred.

### Statistical Analysis

Descriptive statistics are presented as mean (SD), median and interquartile range (IQR), or percentage, as appropriate. We selected nonparametric methods (χ^2^ test, Fisher exact test, and 2-sample Wilcoxon rank sum test) for statistical evaluations. For the primary outcome (ie, the resolution rate), we ran conventional and random-effects logistic regression models (in which the reminder served as the unit of analysis and the patient was the clustering factor). For all other outcomes, the patient served as the unit of analysis. The odds ratios (ORs) derived from logistic regression analyses are presented with their 95% CIs and respective *P* values. All analyses in this RCT followed the intent-to-treat principle. Two-tailed *P* < .05 was considered to indicate statistical significance. Stata, version 15, statistical software was used for analysis (StataCorp LLC).

Given the potential consequences of frequent alert exposure by clinicians, alert fatigue was also analyzed. It is a condition in which a health care professional, after having been exposed to too many notifications, develops a defensive attitude against alerts, usually ignoring or overriding them.^31^ Alert fatigue was measured by testing for the effects of time and the interaction between group and time, looking for changes in patterns related to resolution rates. The interaction between group and time to assess was used if the intervention effect was subject to alert fatigue over time (ie, desensitizing response to alerts).

## Results

When the interim analysis was conducted (July 15, 2016), the prespecified final sample of generated reminders had already been reached (ie, 6397 reminders, with an overall resolution rate of 25.3% [1618 of 6397]). At that point, 2390 patients had been randomized (1198 to the intervention group and 1192 to the control group). The CDSS triggered 2.68 guidance messages per individual on average, a largely unexpected result that can be attributed to technical and coding improvements in the interplay between the EHRs and the CDSS. We decided not to stop the trial to explore whether the intervention was subject to a decay effect attributable to alert fatigue, thereby increasing power in the analyses of secondary outcomes. The end date was set to December 31, 2016.

From November 1, 2015, to December 31, 2016, a total of 20 563 patients were admitted to the hospital. The study population consists of 6480 patients (31.5%) admitted to the internal medicine wards of Vimercate Hospital (Figure). Patients were randomized either to the intervention group (n = 3242), in which CDSS-generated reminders were displayed to physicians, or to the control group (n = 3238), in which reminders were generated but not shown. The study groups were well balanced with regard to sex, age, and other baseline characteristics (Table 1). The mean (SD) age of patients was 70.5 (17.3) years, and 54.5% (3532 of 6480) were men. The mean (SD) number of medications for chronic diseases per patient was 9.09 (5.97). Most patients were admitted to the hospital for reasons primarily associated with cardiovascular (28.9% [1875 of 6480]) or respiratory (20.4% [1323 of 6480]) diseases.

**Figure.**
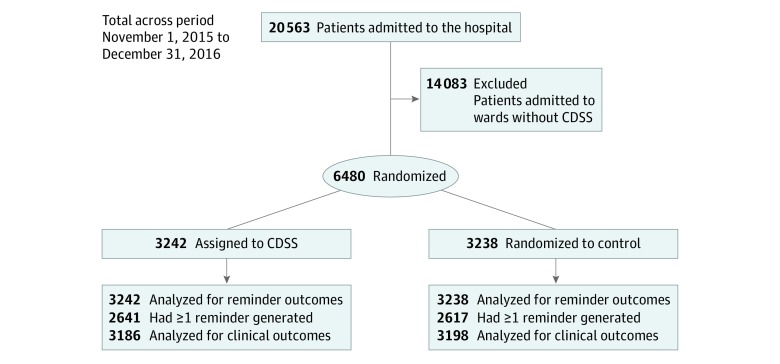
CONSORT Diagram of the Trial Timeline Shown are hospitalized and randomized patients in the observation period. CDSS indicates clinical decision support system.

**Table 1.** Baseline Demographic and Clinical Characteristics and Reasons for Admission by Study Group

Variable	No. (%)	
	Intervention (n = 3242)	Control (n = 3238)
**Demographic Data**		
Male	1766 (54.5)	1766 (54.5)
Age, mean (SD), y	70.5 (17.3)	70.6 (17.3)
Age group, y		
0-30	126 (3.9)	123 (3.8)
31-60	630 (19.4)	632 (19.5)
61-80	1452 (44.8)	1453 (44.9)
>80	1034 (31.9)	1030 (31.8)
**Clinical Status**		
Blood pressure, mean (SD), mm Hg		
Systolic	126 (16)	125 (16)
Diastolic	68 (9)	68 (9)
BMI, mean (SD)^a^	25.90 (5.56)	25.64 (5.32)
Medications for chronic diseases, mean (SD), No.	9.16 (6.08)	9.01 (5.85)
***ICD-9* Chapter and Category**		
Infectious diseases		
Sepsis	82 (2.5)	77 (2.4)
Others	48 (1.5)	47 (1.5)
Total	130 (4.0)	124 (3.8)
Malignant neoplasia		
Lung cancer	44 (1.4)	63 (1.9)
Metastatic to respiratory and digestive system	47 (1.4)	25 (0.8)
Others	159 (4.9)	202 (6.2)
Total	250 (7.7)	290 (9.0)
Endocrine system		
Electrolyte alterations	20 (0.6)	33 (1.0)
Type 1 and type 2 diabetes	6 (0.2)	7 (0.2)
Others	20 (0.6)	14 (0.4)
Total	46 (1.4)	54 (1.7)
Hematological	27 (0.8)	41 (1.3)
Psychiatric	39 (1.2)	37 (1.1)
Neurological	67 (2.1)	66 (2.0)
Heart diseases		
Heart failure	336 (10.4)	345 (10.7)
Myocardial ischemia	188 (5.8)	183 (5.7)
Arrhythmias	90 (2.8)	91 (2.8)
Angina pectoris	31 (1.0)	27 (0.8)
Others	47 (1.4)	44 (1.4)
Total	692 (21.3)	690 (21.3)
Vascular		
Stroke	136 (4.2)	133 (4.1)
Complications of stroke	37 (1.1)	28 (0.9)
Brain hemorrhage	24 (0.7)	19 (0.6)
Others	56 (1.7)	60 (1.9)
Total	253 (7.8)	240 (7.4)
Respiratory		
Pneumonia	235 (7.2)	244 (7.5)
Chronic obstructive pulmonary disease	50 (1.5)	55 (1.7)
Pleurisy	34 (1.0)	38 (1.2)
Bronchitis	42 (1.3)	37 (1.1)
Asthma	13 (0.4)	10 (0.3)
Calcified pulmonary nodules	235 (7.2)	225 (6.9)
Others	59 (1.8)	46 (1.4)
Total	668 (20.6)	655 (20.2)
Gastrointestinal		
Liver cirrhosis	60 (1.9)	46 (1.4)
Gallstones	28 (0.9)	23 (0.7)
Intestinal occlusion	18 (0.6)	26 (0.8)
Pancreatic diseases	21 (0.6)	22 (0.7)
Others	105 (3.2)	124 (3.8)
Total	232 (7.2)	241 (7.4)
Renal		
Acute renal insufficiency	56 (1.7)	66 (2.0)
Chronic renal insufficiency	23 (0.7)	35 (1.1)
Stones	16 (0.5)	18 (0.6)
Infections of lower urinary tract	55 (1.7)	55 (1.7)
Others	54 (1.7)	49 (1.5)
Total	204 (6.3)	223 (6.9)
Pregnancy	46 (1.4)	40 (1.2)
Dermatological	25 (0.8)	22 (0.7)
Rheumatologic and orthopedic		
Connective tissue diseases	29 (0.9)	30 (0.9)
Osteoarthrosis	28 (0.9)	20 (0.6)
Femoral neck fracture	56 (1.7)	82 (2.5)
Others	155 (4.8)	136 (4.2)
Total	268 (8.3)	268 (8.3)
Others	141 (4.3)	114 (3.5)
Missing	154 (4.8)	133 (4.1)

Abbreviations: BMI, body mass index (calculated as weight in kilograms divided by height in meters squared); *ICD-9*, *International Classification of Diseases*, *Ninth Revision.*

^a^ Data available for 2641 of 6480 patients (40.8%).

During the study period, 28 394 total reminders were generated (median, 3 reminders per patient per hospital stay; IQR, 1-6). These messages led to a change in practice in approximately 4 of 100 patients. Of these, 17 630 (62.1%) were EBMEDS clinical reminders, 8473 (29.8%) were for drug interactions (6414 for class C interactions and 2059 for class D interactions), and 18 (0.1%) for were for VTE prevention. Overall, 2273 reminders (8.0%) were drug dose reduction warnings in patients with renal failure (Figure). Table 2 lists examples of the most frequently activated guidance.

**Table 2.** Examples of Patient-Specific Clinical and Diagnostic Guidance^a^

Patient-Specific Guidance	Action Required
Interpreting abnormal LDL cholesterol results in type 2 diabetes based on the LDL level and the presence of arterial atherosclerotic disease	Consider drug, consider test
Suggesting hemochromatosis based on increased ferritin, iron, or transferrin saturation results	Consider diagnosis
Recommending to avoid a drug included in the European list of potentially inappropriate medications for an elderly patient^32^	Reconsider drug
Providing advice for prevention of constipation when initiating a strong opioid	Consider drug, change drug
Reminding of missing or outdated follow-up tests for a patient receiving amiodarone hydrochloride	Consider test, consider imaging
Recommending to document or investigate the reason for a decreased glomerular filtration rate in case no structured documentation is found	Missing diagnosis, consider test

Abbreviation: LDL, low-density lipoprotein.

^a^ Classified based on the Evidence-Based Medicine Electronic Decision Support reminder classification thesaurus, with actions potentially required to resolve the detected problem. Examples were selected among 25 top-ranked frequently generated and resolved guidance messages.

### Primary Outcome

Of the 28 394 reminders generated, 10 183 were resolved during the course of the trial (overall resolution rate, 35.9%; 95% CI, 35.3%-36.4%). In total, 5475 of 14 403 reminders were addressed in the intervention group (resolution rate, 38.0%; 95% CI, 37.2%-38.8%) vs 4708 of 13 991 reminders in the control group (resolution rate, 33.7%; 95% CI, 32.9%-34.4%) (Table 3). The corresponding crude OR was 1.21 (95% CI, 1.15-1.27; *P* < .001), whereas the random-effects OR was 1.21 (95% CI, 1.11-1.32; *P* < .001).

**Table 3.** Reminders Generated and Corresponding Resolution Rates (Primary Outcome)^a^

Variable	Intervention (n = 3242)	Control (n = 3238)	Value (95% CI)	*P* Value
Reminders generated, No.	14 403	13 991	NA	NA
Reminders generated per patient, mean (SD)^b^	4.44 (4.94)	4.32 (4.70)	NA	.65
Reminders resolved per patient, mean (SD)^c^	1.69 (2.99)	1.45 (2.66)	NA	.007
Reminders resolved, No.	5475	4708	NA	NA
Reminders resolved, random-effects OR	NA	NA	1.21 (1.11-1.32)	<.001
Reminder resolution rate difference, %	NA	NA	4.4 (3.2-5.5)	<.001
Time to reminder resolution, mean (SD), d^d^	5.2 (13.4)	5.6 (14.0)	NA	<.001

Abbreviations: IQR, interquartile range; NA, not applicable; OR, odds ratio.

^a^ Nonparametric statistical tests (Pearson χ^2^ test and 2-sample Wilcoxon rank sum test) were used for the comparisons between the groups.

^b^ Reminders generated per patient were a median of 3 (IQR, 1-6) for the intervention group and 3 (IQR, 1-6) for the control group.

^c^ Reminders resolved per patient were a median of 0 (IQR, 0-2) for the intervention group and 0 (IQR, 0-2) for the control group.

^d^ Time to reminder resolution was a mean of 2.2 (IQR, 0.9-6.0) days for the intervention group and 2.9 (IQR, 1.0-6.1) days for the control group.

The time to resolution of the reminders was shorter in the intervention group (mean [SD], 5.2 [13.4] days) compared with the control group (mean [SD], 5.6 [14.0] days) (*P* < .001). These results are summarized in Table 3.

### Secondary Outcomes

#### Types of Reminders

The resolution rate was consistent and statistically significant across the different types of reminders. These included EBMEDS clinical reminders (crude OR, 1.21; 95% CI, 1.14-1.29; *P* < .001; random-effects OR, 1.22; 95% CI, 1.12-1.32; *P* < .001), reminders for drug interactions (crude OR, 1.30; 95% CI, 1.19-1.42; *P* < .001; random-effects OR, 1.40; 95% CI, 1.16-1.68; *P* < .001), reminders for class C drug interactions (crude OR, 1.28; 95% CI, 1.16-1.42; *P* < .001; random-effects OR, 1.35; 95% CI, 1.11-1.64; *P* = .003), and reminders for class D drug interactions (crude OR, 1.48; 95% CI, 1.10-1.99; *P* = .009; random-effects OR, 1.34; 95% CI, 1.13-1.60; *P* = .001).

#### In-Hospital All-Cause Mortality

The rate of in-hospital all-cause mortality was similar between the study groups (5.9% [192 of 3242] vs 6.2% [202 of 3238]; OR, 0.95; 95% CI, 0.77-1.17; *P* = .59). After discharge, the rates did not differ between groups at 30 days and 90 days (Table 4).

**Table 4.** Morbidity and Mortality End Points (Secondary Outcomes) by Study Group

Variable	No. (%)		OR (95% CI)	*P* Value
	Intervention (n = 3242)	Control (n = 3238)		
VTE-related in-hospital events	27 (0.8)	27 (0.8)	1.00 (0.56-1.77)	.99
In-hospital all-cause deaths	192 (5.9)	202 (6.2)	0.95 (0.77-1.17)	.59
Deaths within 30 d after discharge	364 (11.2)	379 (11.7)	0.95 (0.82-1.11)	.55
Deaths within 90 d after discharge	536 (16.5)	557 (17.2)	0.95 (0.84-1.09)	.47

Abbreviations: OR, odds ratio; VTE, venous thromboembolism.

#### VTE-Related Mortality

Of 54 patients with VTE-related in-hospital events (27 in each study group), none died during hospitalization (Table 4). Three patients died in the month after hospital discharge, and 6 patients (3 in the intervention group) died within 90 days of hospital discharge. The resolution rate was higher in the intervention group (22.2% [6 of 27]) compared with the control group (0% [0 of 27]).

#### Length of Hospital Stay

The length of hospital stay did not differ between the study groups, with a median time of 8 days (IQR, 5-13 days) for the intervention group and a median time of 8 days (IQR, 5-14 days) for the control group (*P* = .36). Data for clinical outcomes (in-hospital all-cause mortality, in-hospital morbidity for VTE-related causes, and the length of hospital stay) were missing for 96 of 6480 patients (1.5% of the study population), with no clinically or statistically significant differences between the study groups.

### Alert Fatigue

Although the resolution rate increased over time, alert fatigue did not change. Specifically, the resolution rate increased from 25.3% (1618 of 6397) during the first trial period (results of the interim analysis) to 38.9% (8557 of 21 997) during the second trial period (*P* < .001), for an overall resolution rate of 35.9%. In contrast, alert fatigue, as measured by testing for the effects of time and the interaction between group and time, was unchanged during the trial. Results showed that the intervention effect was not subject to alert fatigue over time (*P* = .47 for the interaction between group and time).

## Discussion

An international, vendor-based CDSS that was embedded in the EHRs of a general hospital (Vimercate Hospital) to provide real-time guidance slightly reduced potentially inappropriate care compared with access to evidence summaries as a part of usual care through manual searching of point-of-care medical information summaries. The EBMEDS activated a median of 3 reminders per patient per hospital stay, identifying situations at risk for inappropriate medical orders. These messages led to a change in practice in approximately 4 of 100 patients. Although the improvement was small, it was consistent across all types of guidance messages. Although alert fatigue did not have a major effect on CDSS use, resolution rates increased over time. The small effect on resolution rate might be attributed to the large volume of clinically inconsequential alerts; even if alerts were associated with patient characteristics and integrated with patient-specific variables (eg, alerts for nephrology dose reduction warnings were triggered only for high-risk patients), the CDSS was unable to efficiently filter guidance messages. Overall, the CDSS directed approximately 14 400 messages to physicians allocated to the intervention group, 13 600 (94.7%) of which did not lead to a change in care. A dynamic selection of alerts based on their perceived relevance may increase their ability to alter practice.

We ensured that the intervention, EBMEDS, a commercial CDSS covering a wide array of health conditions across specialties, was integrated into the hospital EHRs to generate patient-specific recommendations in a real-world hospital characterized by a high mortality rate (approximately 16.9% [1093 of 6480] at 90 days in our RCT). However, most of the processes of care that were targeted were not strongly associated with mortality, and our study was underpowered to detect a difference in mortality. This is consistent with the wider evidence base showing CDSSs to be more effective at changing practice than patient outcomes.^4^ Critical laboratory results, transfers to the intensive care unit, administration of antidotes (eg, naloxone hydrochloride to reverse excessive doses of opiate analgesia), and other intermediate harm outcomes would have provided additional key information to better understand the effectiveness of CDSSs in improving health care outcomes. The present study was not designed to target life-threatening situations in which a timely message could save a life.

Automated identification of patient problems can enhance clinical performance by offsetting repetitive or monitoring activities, thus freeing physicians to focus on more demanding and sophisticated tasks.^33^ However, automation is not without drawbacks and must be managed, particularly when involving additional data entry. For example, algorithms requiring physicians to evaluate several risk factors or test results present additional workload. Poor automation can be costly or futile. Presenting alerts after medications are selected might be too late, with most of the work of prescribing occurring before using the computer.^34^

Traditionally, hospital software has served a limited range of medical specialties with a narrow focus (eg, antibiotic prescription) and has been built on a platform of locally developed databases that may present challenges to the integration, updating, and reliability of information to guide decision-making. We studied a data model consisting of clinical measures and international standards for the management of health problems. An international publisher (Duodecim Medical Publications Ltd, owned by the Finnish Medical Society) filtered the medical evidence available to physicians for relevance and reliability. Our findings support the idea that advanced CDSSs can be assimilated into a turnkey service.^35^ This type of CDSS could cover a wide spectrum of diseases and interventions, aggregating several databases that could be selected based on individual hospital needs.

### Comparison With Other Studies

Previous randomized and nonrandomized trials that assessed the consequences of similar interventions targeting health care professionals (automatic provision of decision support) showed substantial positive results.^36,37^ To date, CDSS interventions have been designed to target patients with specific conditions, often those with poor control of their chronic illness, or in problematic settings, such as potentially inappropriate prescribing.^38^ The number of implemented automated rules in several studies included in those systematic reviews was often less than 10. In contrast, our RCT showed a clinically important change in targeted prescribing among patients with multimorbidity through the use of a set of 262 clinical support rules predefined by the publishing company (Duodecim Medical Publications Ltd). The first RCT^30^ that tested EBMEDS measured the change in the number of messages triggered over 12 months in a Finnish primary care setting, which did not show a difference between randomization groups. However, important technical and clinical issues, including a large proportion of healthy adults whose EHRs did not trigger any guidance message, compromised the trial. In addition, results from PRIMA-EDS (Polypharmacy in Chronic Diseases–Reduction of Inappropriate Medication and Adverse Drug Events in Older Populations by Electronic Decision Support),^39^ a cluster RCT in primary care, were reported in 2016. Additional trials testing commercial CDSSs are needed to explore whether improved alignment with recommendations is a consistent finding and to compare different types of CDSSs, particularly those based on artificial intelligence rather than on evidence selected through human oversight. Important areas of future research include identifying the most effective guidance messages that physicians are likely to regularly consult, understanding if changes in behavior and safety can modify patient outcomes, and investigating the influence of cross-cultural differences. The role of CDSSs for improving prescribing safety in combination with other interventions, such as continuing medical education, should also be explored.^40^

### Policy Implications

Technology companies are expected to increase their investment in the health care sector through the development of artificial intelligence and machine learning to support clinical practice.^41^ Drawn by the prospect of large profits, the arrival of several CDSS vendors on the market may be associated with exaggerated reports on the effectiveness of these products for improving clinical practice, which fail to consider the quality of the guidance messages or rules and the way in which they have been developed. Preliminary experience with CDSSs precipitated concerns regarding their accuracy because the technology, for example, suggested wrong treatments that exposed patients to potential harms in some instances.^42^ As substantial a priori trust in the technology by clinicians is to be expected, it is crucial to assess the relevance and validity of CDSS guidance messages, particularly in terms of safety. However, CDSSs are not likely to be assessed in controlled settings unless a regulation process similar to that leading to food and drug approval is enforced. The legal constraints and acceptability of widespread algorithm use require careful consideration. Quality indicators that can be used to evaluate CDSSs should be developed and may inform clinicians and policy makers, who must choose the most appropriate product. Some failure and inefficiencies should be expected as CDSSs evolve through an iterative approach.

### Strengths and Limitations

This study has strengths and limitations. The pragmatic design of this RCT and the enrollment of most patients admitted to the internal medicine wards of a large Italian general hospital increase the generalizability of the results. However, all 262 clinical support rules were implemented simultaneously. This approach may not be representative of the expected day-to-day operation of a CDSS that is integrated into an existing EHR of a typical institution. Because physicians were assigned patients regardless of their randomization, the same physician may have cared for individuals in the intervention group and the control group. Therefore, care for patients in the control group may have been influenced by reminders that physicians were exposed to for their patients in the intervention group, diluting the differences between study arms. Contamination might have biased the results toward a more conservative effect estimate.^43^

Vimercate Hospital did not use an explicit process for identifying potentially inappropriate reminders or clinical suggestions but rather accepted at face value what was proposed by EBMEDS. A more in-depth evaluation of the clinical workflow, user preferences, and alert local validation may have increased alert resolution rates.^44^ Many factors likely account for the stable proportion of alerts resolved over time in our RCT, including the use of a simple intervention, increased familiarity with the CDSS, and the popularity or acceptance of alerts within local workplaces and the broader culture of health care practice. Also, detecting a potentially dangerous drug combination does not automatically translate into an existing risk for patients and cannot be categorized as a genuine medical error.^45^

## Conclusions

This pragmatic RCT provides evidence that an international commercial CDSS intervention was marginally effective at modifying prescribing behavior and the quality of care, although the change did not statistically significantly affect patient outcomes. Absolute effects on improving adherence to recommendations were limited, affecting approximately 4 in 100 patients. The minimum requirements for implementing CDSSs include the following: an EHR featuring a common semantic, formatting that can manage complex health data,^46,47,48^ and the ability to integrate data to either a vendor-based or an open-source CDSS.^49,50^ These key components are becoming common preconditions to CDSS use in hospitals and can encourage and sustain the adoption of CDSSs in health care systems.

## Notes

Supplement 1. **Trial Protocol**

Supplement 2. **Data Sharing Statement**
