# Supplementary material for: Effectiveness of a Hospital-Based Computerized Decision Support System on Clinician Recommendations and Patient Outcomes: A Randomized Clinical Trial
Source: JAMA Netw Open. 2019 Dec 11;2(12):e1917094. doi: 10.1001/jamanetworkopen.2019.17094 (PMC6991299; doi:10.1001/jamanetworkopen.2019.17094)
Supplement: Supplement 1. — Trial Protocol [file jamanetwopen-2-e1917094-s001.pdf]

Implementing an evidence-based computerized decision support system to improve patient care in a general hospital: the CODES study protocol for a randomized controlled trial.

Lorenzo Moja (1,2) < corresponding author: lorenzo.moja@unimi.it >

Hernan Polo Friz (3) < hernanemilio.polofriz@aovimercate.org >

Matteo Capobussi (4) < matteo.capobussi@gmail.com >

Koren Kwag (2) < kkwag12@gmail.com>

Rita Banzi (5) < rita.banzi@marionegri.it >

Francesca Ruggiero (2) < francesca.ruggiero@unimi.it >

Marien González-Lorenzo (1) < mariengonzalezlorenzo@gmail.com >

Elisa Giulia Liberati (6) < egl3@le.ac.uk>

Massimo Mangia (7) < m.mangia@medilogy.it >

Peter Nyberg (8) < peter.nyberg@duodecim.fi >

Ilkka Kunnamo (8) < ilkka.kunnamo@duodecim.fi >

Claudio Cimminiello (3) < claudio.cimminiello@gmail.com>

Giuseppe Vighi (3) < giuseppedanilo.vighi@aovimercate.org >

Jeremy Grimshaw (9) < jgrimshaw@ohri.ca >

Stefanos Bonovas (10) < sbonovas@gmail.com >

(1) Department of Biomedical Sciences for Health, University of Milan, Via Pascal 36, 20133 Milan, Italy

(2) Clinical Epidemiology Unit, IRCCS Orthopedic Institute Galeazzi, Via Galeazzi 4, 20161 Milan, Italy

(3) Internal Medicine Division, Medical Department, Vimercate Hospital, Via Santi Cosma e Damiano 10, 20871 Vimercate, Italy

(4) School of Specialization in Hygiene & Preventive Medicine, University of Milan, Milan, Italy

(5) IRCCS Mario Negri Institute for Pharmacological Research, Via La Masa 19, 20156 Milan, Italy

(6) Department of Health Science, University of Leicester, Centre for Medicine, University Road, Leicester LE1 7RH, UK

(7) Medilogy Srl, Viale Monza 133, 20125 Milan, Italy

(8) Duodecim Medical Publications Ltd, Kaivokatu 10 A, 00101 Helsinki, Finland

(9) Clinical Epidemiology Program, Ottawa Hospital Research Institute & Department of Medicine, University of Ottawa, 501 Smyth Road, Ottawa, ON K1H 8 L6, Canada

(10) Humanitas Clinical and Research Center, Via Manzoni 56, 20089 Rozzano, Milan, Italy

Abstract

Background: Computerized decision support systems (CDSSs) are information technology-based software that provide health professionals with actionable, patient-specific recommendations or guidelines for disease diagnosis, treatment, and management at the point-of-care. These messages are intelligently filtered to enhance the health and clinical care of patients. CDSSs may be integrated with patient electronic health records (EHRs) and evidence-based knowledge.

Methods/Design: We designed a pragmatic randomized controlled trial to evaluate the effectiveness of patient-specific, evidence-based reminders generated at the point-of-care by a multi-specialty decision support system on clinical practice and the quality of care. We will include all the patients admitted to the internal medicine department of one large general hospital. The primary outcome is the rate at which medical problems, which are detected by the decision support software and reported through the reminders, are resolved (i.e. resolution rates). Secondary outcomes are resolution rates for reminders specific to venous thromboembolism (VTE) prevention, in-hospital all causes and VTE-related mortality, and the length of hospital stay during the study period.

Discussion: The adoption of CDSSs is likely to increase across healthcare systems due to growing concerns about the quality of medical care and discrepancy between real and ideal practice, continuous demands for a meaningful use of health information technology, and the increasing use of and familiarity with advanced technology among new generations of physicians. The results of our study will contribute to the current understanding of the effectiveness of CDSSs in primary care and hospital settings, thereby informing future research and health care policy questions related to the feasibility and

value of CDSS use in health care systems. This trial is seconded by a specialty trial randomizing patients in an oncology setting (ONCO-CODES, ClinicalTrials.gov: NCT02645357).

Trial registration: ClinicalTrials.gov: NCT02577198

Keywords

Computerized decision support systems, electronic health records, evidence-based medicine, pragmatic trial, randomized controlled trial, reminder systems.

Background

Background and rationale

Despite the proliferation of clinical guidelines and continued efforts by local and national health care systems to optimize decision-making on patient diagnosis, treatment, and management, the quality of medical care is variable and often suboptimal [1]. There remains an apparent discrepancy between the growing availability of scientific evidence and the application of this evidence into medical care [2,3]. Non-adherence to evidence-based guidelines, medical errors, and omissions in everyday practice may occur because of time pressure, inexperience, reliance on memory, multitasking, and failures in healthcare team coordination.

Computerized decision support systems (CDSSs) are information technology-based software that provide health professionals with actionable, patient-specific recommendations or guidelines for clinical care at the point-of-care; these messages are intelligently filtered and presented at appropriate times during the decision-making process in order to enhance patients' health [4,5]. The opportunity to improve patient care by increasing clinicians' accessibility to medical knowledge at the site of practice represents one of the main incentives for investing in the development and evaluation of these sophisticated information systems.

In particular, studies focusing on the effectiveness of "new generation" CDSSs demonstrate their potential to assist with problems raised in clinical practice, decrease the rate of medication errors, increase clinicians' adherence to guideline- or protocol-based care, and, ultimately, improve the overall efficiency and quality of health care delivery systems [6-19]. These innovative systems can be integrated into hospital electronic health records (EHRs) and feature authoritative point-of-care information services and evidence-based knowledge [20].

This has led to some early work: a systematic review assessing the effectiveness of such "new generation" CDSSs demonstrated encouraging results [21]. Although this review did not show CDSSs to affect mortality, they were shown to moderately improve morbidity outcomes. Differences were further observed for costs and health services utilization, but these were often inconsistent in the direction of effect and small in magnitude. The conclusion of a landmark paper, published nearly 15 years ago, still reflects the current scenario: *"Although the promise of clinical decision support system-facilitated evidence-based medicine is strong, substantial work remains to be done to realize the potential benefits"* [22].

Current research on CDSSs suffers two noteworthy limitations [5]. First, while numerous studies have evaluated the effectiveness of CDSSs, comparatively few implemented a randomized controlled trial (RCT) design. Second, most published evaluations of the impact of CDSSs on health care quality were conducted in academic medical centers using "homegrown" systems that featured restricted clinical content for particular conditions (e.g., thromboprophylaxis). There is limited research on "mature" CDSSs that are commercially available and capable of supporting a wide range of clinical activities. Across countries, the adoption of these systems by hospitals is likely to increase in the future.

Objective

Our study aims to evaluate the effectiveness of patient-specific, point-of-care reminders generated by the Medilogy Decision Support System (MediDSS) [23] on clinical practice and the quality of care in a general hospital.

Methods/Design

This protocol is reported in accordance with the SPIRIT 2013 guidance for content of clinical trial protocols [24,25].

Trial design

The CODES (COmputerized DEcision Support) trial will implement a pragmatic, parallel group, and randomized controlled design with 1:1 allocation ratio. The flow diagram of the study can be found in Figure 1.

Study setting

The study will involve the medical staff of the internal medicine departments of the Vimercate Hospital from Azienda Ospedaliera di Desio e Vimercate (AODV) a multi-site hospital system located in the Lombardy region of Italy [26]. AODV includes hospitals and health units distributed in the Province of Monza and Brianza, which covers a population of approximately 850,000 inhabitants. The Vimercate Hospital has a medical staff of more than 230 doctors, a total of over 900 health professionals, and an overall capacity of 489 beds. It supports over 20 specialties and subspecialties. The catchment population is approximately 200,000 inhabitants, with more than 15,000 admissions per year. In addition to the in-patient wards, the hospital houses many facilities for diagnosis and treatment.

Since 2010, the Vimercate Hospital has been electronically tracking all clinical and administrative information through an EHR system based on the *"Tabula Clinica"* platform (developed by Dedalus S.p.A.) [27].

Eligibility criteria and recruitment

As a pragmatic clinical trial [28,29], CODES seeks to investigate the effectiveness of MediDSS reminders in everyday clinical practice with diverse patients and varying conditions. Thus, we will enroll all of the patients admitted into the internal medicine departments of the AODV, without applying any exclusion criteria.

Intervention

We selected the Medilogy Decision Support System (MediDSS) after a comparative assessment of available editorial products using a predefined set of essential criteria [30,31]. MediDSS is a product by Medilogy, an Italian developer of scientific software and medical technology. Medilogy translated and adapted Evidence-Based Medicine electronic Decision Support (EBMeDS) [32], a CDSS developed by Duodecim Medical Publications Ltd., a company owned by the Finnish Medical Society Duodecim. EBMeDS can be described as a set of rules (scripts) based on EBM guidelines and applied to structured health data. MediDSS further includes knowledge from Swedish, Finnish, INteraction X-referencing (SFINX), a drug-drug interaction database containing concise evidence-based information about the harms and benefits of about 18,000 drug interactions and adverse events [33].

MediDSS may be used as a stand-alone application, or may integrate structured patient data from EHR to generate patient-specific reminders, therapeutic suggestions, and diagnosis-specific links to full-text guidelines. Reminders are automatically generated and displayed on the monitors of clinicians when they open a patient's EHR, enter a new diagnosis, prescribe a drug, or when new laboratory test results are available. Reminders were formed using international evidence-based guidelines and subsequently approved by an international panel of experts. Our study will use international reminders (n=262) that cover a large number of health conditions across specialties and are derived from the EBMeDS and SFINX database. In addition, 17 local reminders have been carefully selected by a team of doctors at Vimercate hospital, along with members of the trial team. Table 1 reports some examples of the reminders. Figures 2 and 3 show a snapshot of the activation button and of the actual reminders.

MediDSS reminders will be shown on the EHR of patients only within the intervention group. During the care of control group patients, the generated reminders will not be shown to the physicians, such that the control is usual clinical practice without the use of the MediDSS service. However, physicians

in both groups will have access to the best evidence for usual care at all times during the trial through the active searching of full-text EBM guidelines on Internet. All participating physicians will be informed on the availability and use of the MediDSS system.

Stepped wedge implementation

The intervention is a new technology: its integration in the current hospital system requires the configuration and customization of the software. To allow security controls and successful implementation, the CDSS will be sequentially rolled out to participants over a number of time periods. We anticipate that the number of periods will be limited (i.e. two or three periods). Over an initial period, all participants will receive the intervention. The order in which participants will receive the intervention is not determined at random, but will be determined by selecting physicians prone to provide constructive feedback to the implementation team. The RCT adopts a stepped wedge implementation of the intervention, but not a stepped wedge design [34]. Sequential roll-out of the intervention will not be considered a pilot phase of the trial, but a part of the whole RCT.

Selection and development of priority reminders

In order to encourage the participation of the hospital staff within the study, we invited hospital representatives to assess the priority needs of the hospital wards and develop a set of reminders to address them. One topic of particular interest to the hospitals involved venous thromboembolism (VTE) prevention. The rationale for the prioritization of this condition is provided below:

(i) Despite evidence supporting the benefits of VTE prophylaxis based on the risk stratification process [35] as well as the availability of local hospital guidelines, the prophylactic drugs were inconsistently administered among patients.

(ii) The Hospital of Vimercate has an increasingly large population of elderly (aged > 65 years old) and very elderly (aged > 80 years old) subjects, who have a higher risk of recurrent VTE and acute pulmonary embolism [36].

(iii) Between 2010 and 2012, 45 patients (9.6%, 95% CI: 7.2–12.6) and 75 patients (16.0%, 95% CI: 12.9–19.5) died, respectively, within 30 days and 90 days after discharge due to VTE [36].

(iv) Research has shown the use of CDSSs to improve the assessment of patients' risk for VTE, facilitate appropriate administration of prophylaxis interventions, and reduce the rate of symptomatic VTE in hospitalized patients [37-53].

In order to develop the set of reminders for VTE prevention, the local hospital expert group proposed a risk stratification process based on the Padua Score [54]. This formula calculates the overall risk (low or high) of VTE for each patient using both clinical and surgical risk factors. Details of the underlying algorithm are provided in Box 1.

Besides the VTE prevention therapy reminders, other scripts were chosen for development and tailored to the hospital's needs. The following alerts were identified by the hospital's clinicians as of special interest:

– Adjusting warfarin dose in atrial fibrillation,

– Alert on heparin-induced thrombocytopenia,

– Clopidogrel, prasugrel or ticagrelor and aspirin in acute ST-segment elevation myocardial infarction (STEMI),

– Low-molecular-weight heparin as anticoagulant for patients with venous thromboembolism and cancer,

– Selection of antithrombotic therapy in atrial fibrillation on the basis of the CHA2DS2VASc score,

– Supplementary laboratory measurements in warfarin therapy,

– Low-dose aspirin: dosing in renal insufficiency,

– Drug-drug interaction: aspirin and ACE inhibitor,

– Warfarin and paracetamol: drug-drug interaction,

– ACE inhibitor or sartan for diabetic patients with albuminuria,

– Glimepiride warning in renal insufficiency,

– LDL-cholesterol concentration in patients with Type 2 diabetes,

– High BNP or proBNP; untreated congestive heart failure (CHF)?

– Initial laboratory examinations in patients with congestive heart failure (CHF),

– Beta blockers in the prevention of gastrointestinal bleeding in patients with cirrhosis,

– ACE inhibitors or angiotensin-receptor blockers for patients with diabetes and hypertension but no microalbuminuria.

Qualitative integration

The validity of this RCT relies on the actual implementation of MediDSS by physicians in their clinical activities. Healthcare service studies on CDSSs, however, consistently suggest that the mere provision of such technology does not guarantee its uptake. In fact, even if a CDSS is readily available within a hospital, clinicians often fail to follow its recommendations, ignoring in some cases up to 96% of its alerts [55]. Given this context, our RCT is informed by qualitative interviews aimed to detect the barriers and facilitators to MediDSS uptake as perceived by diverse health professionals involved in patient care (e.g., physicians and nurses). The interviews are a part of a larger cross-sectional study, which involves three Italian hospitals [56]. The interviews will explore variables that may hinder the use of a CDSS in everyday clinical practice, including technical (e.g., poor usability or knowledge of system), individual (e.g., negative perception of CDSS or EBM, lack of motivation), group or

organizational (e.g., structural or administrative constraints), and cultural factors (e.g., adverse social norms).

When feasible, the trial will be tailored to address the specific needs emerging from the qualitative assessment. We will collect feedbacks about usability, possible errors, or inaccuracies of the information and recommendations provided. We will offer the best possible solutions to clinicians and hospital staff to overcome these problems. We will further organize and facilitate group discussions among participants to address negative perceptions or misleading beliefs about CDSSs. The qualitative study seeks to support the use of CDSS by participants, thus increasing the integrity of the intervention and associated compliance.

Study outcomes

Primary outcome: the rate at which the medical problems, which are detected by the MediDSS software and reported through the reminders, are resolved (i.e. resolution rates).

Secondary outcomes: (i) resolution rates for the VTE prevention reminders, (ii) in-hospital all causes mortality (iii) VTE-related mortality, (iv) in-hospital morbidity for VTE-related causes, and (v) the length of hospital stay during the study period.

Sample size

We calculated the sample size on the basis of the primary outcome. A previous systematic review assessing the effects of computer reminders delivered to clinicians at the point-of-care on health care processes and outcomes found a median improvement of 4.2% in process adherence across all reported process outcomes [57]. Accordingly, assuming resolution rates of 5% in the intervention group versus 3% in the control group due to a possible group contamination, we calculated that a sample of 4,230 reminders will be necessary to detect the difference between the two groups (power = 0.90; α = 0.05,

two-sided; 1:1 allocation). Because estimates for intracluster correlation are not available, we increased the required sample size (by 10%) to 4,650 reminders to account for clustering by patient.

Moreover, based on a prior study evaluating EBMeDS, which recorded an average of 0.30 reminders per individuals triggered at baseline [58], we determined that a total number of 15,500 patients (7,750 per group) need to be enrolled. This figure corresponds to a conservative estimate of the recruitment period of 24 months for the internal medicine departments of the Vimercate Hospital.

Allocation and blinding

Anonymous patient identification (ID) numbers in the EHR system will be the unit of randomization. An individual external to the study group will generate the anonymous IDs using a formula based on patients' unique fiscal code numbers.

We will randomly assign patients to either the control or experimental group with a 1:1 allocation. We will follow a computer generated randomization schedule stratified by gender and age (0–30, 31–60, 61–80, >80 years) using permuted blocks of random sizes [59]. Patients will be randomized immediately after the first launch of their EHR (entry of demographic data by physicians at hospital admission), and the allocation will be maintained through successive admissions.

Patients and study investigators (i.e., researchers, statisticians, information technology specialists, and hospital representatives) will be blinded to the allocation of participants. We will maintain the blinding up to the dataset disclosure. On the other hand, blinding of physicians is not feasible due to the nature of the intervention: the physician will know that a patient has been allocated to the intervention group if an automatic, patient-specific reminder is displayed on the screen.

Data collection

The data collection for this study will follow the standard data collection procedures of the AODV. We will collect demographic (i.e., gender, age) and administrative (i.e., anonymous patient ID, admission and discharge dates, diagnoses) data from the EHR archive on a daily basis. Information on reminders, including all scripts that have been activated in a patient's record, will also be collected daily, but during the night, so as not to disturb or slow down the use of the patient EHR.

Statistical methods

For the primary outcome (i.e., resolution rates), the reminder will serve as the unit of analysis, and the patient the clustering factor. The patient will be the unit of analysis for the secondary outcomes (i.e., length of stay and in-hospital mortality). All analyses will follow the intention-to-treat principle: patients will be analyzed in the group to which they have been randomized. Descriptive statistics will be presented as means ± standard deviations (SD), medians and interquartile ranges (IQR), or percentages when appropriate. We will compare continuous variables using the Student's *t*-test when normally distributed, and the non-parametric two-sample Wilcoxon rank-sum (Mann-Whitney) test when they are not normally distributed. We will compare categorical variables using the chi-squared test or the Fisher's exact test, as appropriate. To model the resolution rates of the reminders, we will run a random effects logistic regression analysis, accounting for clustering by patient [60].

For hypothesis testing, we will consider a probability level of less than 0.05 as statistically significant. All statistical tests will be two-sided. We will use the Stata software to perform all statistical analyses (Stata Corp., College Station, TX, USA).

Data monitoring

Data monitoring will inform the CODES trial conduct, identifying the potential need for adjustments:

(i) ***Sample size recalculation****:* Because the sample size calculation utilizes several assumptions, we will analyze the first batch of data collected and adjust the estimated sample size, if necessary, at the end of the sequential roll-out of the intervention. The 24-month recruitment period may also be adjusted, accordingly.

(ii) ***Interim analysis****:* We will perform an interim-analysis on the primary endpoint after 50% of the patients have been randomized, after 50% of the expected events have occurred, or after 12 months of the study's initiation (the assumed half-life of the trial), whichever occurs first. An independent statistician that is blind to the patient allocation will perform the analysis. This analysis will inform whether the intervention has been proven for efficacy (beyond reasonable doubt). We will subsequently decide whether (or not) it is necessary to modify the study or prematurely terminate it, if necessary.

(iii) ***End of trial***: The end of trial will occur thirty days after the randomization of the last EHR. We will submit an End of Trial notification and final report to the competent Ethical Committee, the AODV, and to the Sponsor.

Harms

We do not anticipate any harms (or other unintended effects) to study participants. Intervention and control groups will differ in the presence (intervention) or absence (control) of automatic reminders displayed on physicians' monitors. Patients assigned to the control group will receive usual care without the reminders. Nevertheless, we will consult an External Advisory Board in the event that the discontinuation of the study becomes an option due to unforeseeable reasons.

Ethical and Regulatory considerations

This study is conducted in accordance with the principles of the Declaration of Helsinki (October 2013) [61]. As the CODES trial has a cluster design (several reminders, the unit of analysis, may derive from

the same EHR, the unit of randomisation), we followed the Ottawa statement to identify research participants and apply ethical and regulatory protections [62,63]. The intervention (electronic CDSS reminders) does not directly target patients, but physicians who can be considered as the participants of the study. The risks associated with the participation of physicians in the CODES trial are negligible. Physicians will be fully informed about the involvement of the AODV in the CODES trial and trained to use the intervention. Requiring the signed consent of each physician is not feasible and will impact on the validity and generalizability of study results. Some have argued that healthcare professionals have an obligation to participate in health system or knowledge translation research [64,65]. We consider that the waiver of signed consents will not adversely affect the rights or welfare of the research participants.

Protocol amendments

Any changes to the research protocol that may impact the study conduct (e.g., changes in study design, eligibility criteria, study outcomes, sample size, study procedures, or significant administrative aspects) will require a formal amendment of the protocol. We will communicate any such amendments to the trial registry (ClinicalTrials.gov), and notify the health authorities in accordance with the Italian regulations. We will further seek the approval of the Ethical Committee for any amendments to the protocol.

Confidentiality

The trial staff will ensure the maintenance of participants' anonymity. The participants will be identified only by their initials and anonymous patient ID number. Depersonalised data will be extracted from the EHR. All documents will be stored securely and accessible only by the trial investigators and authorised personnel.

Clinical data collected during the study will only be accessible to the staff at AODV, thus complying with the current medical practice of the Hospitals. The trial investigators external to the Hospitals (statistician, data manager, information technology personnel, etc.) will not have access to any information at the patients' level.

The CODES trial will comply with the Italian Data Protection Act, which requires data to be anonymised as soon as it is practical to do so.

Dissemination policy

The trial results will be posted on ClinicalTrial.gov as well as published in an open access medical journal.

We will further disseminate the study results to the health professionals of AODV who are involved in the study.

Discussion

Strengths and limitations

The CODES trial has several strengths. First, the randomized controlled study design is recognized as the "gold standard" for testing intervention-outcome hypotheses, allowing us to maximize the likelihood that the differences observed between groups are due to the intervention rather than potential confounding factors. Second, the pragmatic design of the study under conditions that mimics the actual use of CDSSs in practice increases the generalizability of the results as well as allows a more accurate estimation of the intervention's true effectiveness. Third, the choice of an intention-to-treat analysis helps to ensure the pragmatic design of the study; in other words, although not all physicians may

adhere to the reminders within the study, we anticipate that the lack of compliance with evidence-based recommendations occurs in everyday practice.

We must note the methodological limitation that physicians will not be blinded to the treatment allocation. When a patient-specific reminder is automatically displayed on the monitors, the physician will know that the particular patient belongs to the intervention group. We are aware that the unit of allocation (i.e. patient) and the lack of physician blinding can lead to possible group contamination as one physician can have both intervention and control group patients; in this case, a physician may apply the knowledge from a reminder generated for an intervention group patient to a control group patient. This possible learning effect (contamination of knowledge) can decrease the trial effect and lead to a more conservative effect estimate (i.e., towards the null). Randomization at the physician level, however, does not eliminate the possibility of contamination as physicians can care for patients across different wards; this level of randomization would, moreover, increase the organizational complexity of the study.

Conclusion

The use of CDSSs in healthcare systems is likely to increase in the near future due to (i) growing concerns about the quality of medical care; (ii) continuous calls for a meaningful use of health information technology; and (iii) increasing use of and familiarity with advanced technology among new generations of physicians.

Through our pragmatic trial, we will contribute to the current research and understanding of the effectiveness of CDSSs in primary care and hospital settings. The results of our study may inform future research and health care policy questions related to the feasibility and value of CDSS use in health care systems.

Trial status

The implementation phase of the study was completed in November 2015, when the CDSS (MediDSS) was fully integrated with the hospital's EHR (Tabula Clinica). Subject recruitment and data collection began in December 2015 in the Vimercate Hospital.

Ethics approval and consent to participate

The Ethical Committee of the Monza and Brianza Province approved the CODES protocol (approval number 0218, March 13th 2015).

Physicians are the participants of the study and are fully informed about the involvement of the AODV in the CODES trial and trained to use the intervention. Individual signed consent is not feasible and could impact on the validity and generalizability of the results. The Ethical Committee considered that the waiver of signed consents do not adversely affect the rights or welfare of the research participants.

Consent for publication section

Not applicable.

Availability of data and supporting materials

Data sets will be made available for research purposes upon request after the end of the study.

Abbreviations

CDSS: Computerized Decision Support System; CODES: COmputerized DEcision Support; EBM: Evidence-Based Medicine; EBMeDS: Evidence-Based Medicine electronic Decision Support; HER: Electronic Health Record; GFR: Glomerular Filtration Rate; IQR: Interquartile Range; MediDSS:

Medilogy Decision Support System; RCT: Randomized Controlled Trial; SD: Standard Deviation; VTE: Venous Thromboembolism.

Competing interests

Lorenzo Moja is employed by IRCCS Galeazzi and Università degli Studi di Milano, which have nonexclusive contracts with commercial publishers to develop or adapt CDSSs based on critically appraised studies and systematic reviews.

Ilkka Kunnamo is the founder and leader of the EBMeDS system for CDSS, published by the Finnish Medical Society Duodecim. Peter Nyberg is a full-time employee of Duodecim.

Massimo Mangia is the Chief Executive Officer of Medilogy.

Sources of financial, material, and other support

The EBMeDS system is developed by Duodecim Medical Publications Ltd., a Finnish company owned by the Finnish Medical Society Duodecim. It is distributed in Italy by Medilogy S.r.l. Neither Duodecim Medical Publications Ltd nor Medilogy S.r.l. finance the CODES trial.

The research project is financially supported by the Italian Ministry of Health (GR-2009-1606736) and by Regione Lombardia (D.R.L. IX/4340 26/10/2012). The funding sources have no role in the design and conduct of the study; collection, management, analysis, and interpretation of the data; preparation, review, or approval of the manuscript; and decision to submit the manuscript for publication.

Authors' contributions

Lorenzo Moja is the principal investigator of the CODES (COmputerized DEcision Support system) trial. All authors contributed to the conception and design of the study. Lorenzo Moja, Matteo

Capobussi, Koren Kwag, Rita Banzi, and Stefanos Bonovas drafted the manuscript. All authors critically revised and gave final approval for publication of the protocol.

Acknowledgements

We thank Tiina Kortteisto, Tuomas Koskela, Minna Kaila, and Marjukka Mäkelä for their comments on the research project.

References

1. Roshanov PS, Fernandes N, Wilczynski JM, Hemens BJ, You JJ, Handler SM, Nieuwlaat R, Souza NM, Beyene J, Van Spall HG, Garg AX, Haynes RB. Features of effective computerised clinical decision support systems: meta-regression of 162 randomised trials. BMJ. 2013;346:f657.
2. Schuster M, McGlynn E, Brook R. How good is the quality of health care in the United States? Milbank Q. 2005;83:843–95.
3. McGlynn EA, Asch SM, Adams J, Keesey J, Hicks J, DeCristofaro A, Kerr EA. The quality of health care delivered to adults in the United States. N Engl J Med. 2003;348:2635–45.
4. Osheroff J. Improving medication use and outcomes with clinical decision support: a step-by-step guide. Chicago, IL: The Healthcare Information and Management Systems Society; 2009.
5. Berner E. Clinical decision support systems: State of the Art. AHRQ Publication No. 09-0069-EF. Rockville, MD: Agency for Healthcare Research and Quality; 2009.
6. Bright TJ, Wong A, Dhurjati R, Bristow E, Bastian L, Coeytaux RR, Samsa G, Hasselblad V, Williams JW, Musty MD, Wing L, Kendrick AS, Sanders GD, Lobach D. Effect of clinical decision-support systems: a systematic review. Ann Intern Med. 2012;157:29–43.

7. Ash JS, McCormack JL, Sittig DF, Wright A, McMullen C, Bates DW. Standard practices for computerized clinical decision support in community hospitals: a national survey. J Am Med Inform Assoc. 2012;19:980–7.
8. Griffey RT, Lo HG, Burdick E, Keohane C, Bates DW. Guided medication dosing for elderly emergency patients using real-time, computerized decision support. J Am Med Inform Assoc. 2012;19:86–93.
9. Romano MJ, Stafford RS. Electronic health records and clinical decision support systems: impact on national ambulatory care quality. Arch Intern Med. 2011;171:897–903.
10. Terrell KM, Perkins AJ, Dexter PR, Hui SL, Callahan CM, Miller DK. Computerized decision support to reduce potentially inappropriate prescribing to older emergency department patients: a randomized, controlled trial. J Am Geriatr Soc. 2009;57:1388–94.
11. Holbrook A, Thabane L, Keshavjee K, Dolovich L, Bernstein B, Chan D, Troyan S, Foster G, Gerstein H; COMPETE II Investigators. Individualized electronic decision support and reminders to improve diabetes care in the community: COMPETE II randomized trial. CMAJ. 2009;181:37–44.
12. Bonnabry P, Despont-Gros C, Grauser D, Casez P, Despond M, Pugin D, Rivara-Mangeat C, Koch M, Vial M, Iten A, Lovis C. A risk analysis method to evaluate the impact of a computerized provider order entry system on patient safety. J Am Med Inform Assoc. 2008;15:453–60.
13. de Lusignan S, Chan T. The development of primary care information technology in the United kingdom. J Ambul Care Manage. 2008;31:201–10.
14. Chaudhry B, Wang J, Wu S, Maglione M, Mojica W, Roth E, Morton SC, Shekelle PG. Systematic review: impact of health information technology on quality, efficiency, and costs of medical care. Ann Intern Med. 2006;144:742–52.

15. Feldstein AC, Smith DH, Perrin N, Yang X, Rix M, Raebel MA, Magid DJ, Simon SR, Soumerai SB. Improved therapeutic monitoring with several interventions: a randomized trial. Arch Intern Med. 2006;166:1848–54.
16. Sequist TD, Gandhi TK, Karson AS, Fiskio JM, Bugbee D, Sperling M, Cook EF, Orav EJ, Fairchild DG, Bates DW. A randomized trial of electronic clinical reminders to improve quality of care for diabetes and coronary artery disease. J Am Med Inform Assoc. 2005;12:431–7.
17. Kaushal R, Shojania KG, Bates DW. Effects of computerized physician order entry and clinical decision support systems on medication safety: a systematic review. Arch Intern Med. 2003;163:1409–16.
18. Tamblyn R, Huang A, Perreault R, Jacques A, Roy D, Hanley J, McLeod P, Laprise R. The medical office of the 21st century (MOXXI): effectiveness of computerized decision-making support in reducing inappropriate prescribing in primary care. CMAJ. 2003;169:549–56.
19. Dexter PR, Perkins S, Overhage JM, Maharry K, Kohler RB, McDonald CJ. A computerized reminder system to increase the use of preventive care for hospitalized patients. N Engl J Med. 2001;345:965–70.
20. Moja L, Banzi R. Navigators for medicine: evolution of online point-of-care evidence-based services. Int J Clin Pract. 2011;65:6–11.
21. Moja L, Kwag KH, Lytras T, Bertizzolo L, Brandt L, Pecoraro V, Rigon G, Vaona A, Ruggiero F, Mangia M, Iorio A, Kunnamo I, Bonovas S. Effectiveness of computerized decision support systems linked to electronic health records: a systematic review and meta-analysis. Am J Public Health. 2014;104:e12–e22.
22. Sim I, Gorman P, Greenes RA, Haynes RB, Kaplan B, Lehmann H, Tang PC. Clinical decision support systems for the practice of evidence-based medicine. J Am Med Inform Assoc. 2001;8:527–34.

23. Medilogy S.r.l., Medilogy Decision Support System (MediDSS). http://www.medilogy.it. Accessed April 2016.
24. Chan AW, Tetzlaff JM, Altman DG, Laupacis A, Gøtzsche PC, Krleza-Jeric K, Hrobjartsson A, Mann H, Dickersin K, Berlin J, Dore C, Parulekar W, Summerskill W, Groves T, Schulz K, Sox H, Rockhold FW, Rennie D, Moher D. SPIRIT 2013 statement: defining standard protocol items for clinical trials. Ann Intern Med. 2013;158:200–7.
25. Chan AW, Tetzlaff JM, Gøtzsche PC, Altman DG, Mann H, Berlin JA, Dickersin K, Hrobjartsson A, Schulz KF, Parulekar WR, Krleza-Jeric K, Laupacis A, Moher D. SPIRIT 2013 explanation and elaboration: guidance for protocols of clinical trials. BMJ. 2013; 346:e7586.
26. Azienda Ospedaliera di Desio e Vimercate. http://www.aodesiovimercate.it. Accessed April 2016.
27. Dedalus S.p.A., Tabula Clinica. http://www.dedalus.eu. Accessed April 2016.
28. Schwartz D, Lellouch J. Explanatory and pragmatic attitudes in therapeutical trials. J Chronic Dis. 1967;20:637–48.
29. Zwarenstein M, Treweek S, Gagnier JJ, Altman DG, Tunis S, Haynes B, Oxman AD, Moher D; CONSORT group; Pragmatic Trials in Healthcare (Practihc) group. Improving the reporting of pragmatic trials: an extension of the CONSORT statement. BMJ. 2008;337:a2390.
30. Banzi R, Cinquini M, Liberati A, Moschetti I, Pecoraro V, Tagliabue L, Moja L. Speed of updating online evidence based point of care summaries: prospective cohort analysis. BMJ. 2011;343:d5856.
31. Banzi R, Liberati A, Moschetti I, Tagliabue L, Moja L. A review of online evidence-based practice point-of-care information summary providers. J Med Internet Res. 2010, 12:e26.
32. Duodecim Medical Publications, Evidence-Based Medicine electronic Decision Support (EBMeDS). http://www.ebmeds.org. Accessed April 2016.

33. Böttiger Y, Laine K, Andersson ML, Korhonen T, Molin B, Ovesjö ML, Tirkkonen T, Rane A, Gustafsson LL, Eiermann B. SFINX-a drug-drug interaction database designed for clinical decision support systems. Eur J Clin Pharmacol. 2009;65:627–33.

34. Brown CA, Lilford RJ. The stepped wedge trial design: a systematic review. BMC Med Res Methodol. 2006;6:54.

35. Ageno W, Squizzato A, Ambrosini F, Dentali F, Marchesi C, Mera V, Steidl L, Venco A. Thrombosis prophylaxis in medical patients: a retrospective review of clinical practice patterns. Haematologica. 2002;87:746–50

36. Polo Friz H, Molteni M, Del Sorbo D, Pasciuti L, Crippa M, Villa G, Meloni DF, Primitz L, Galli A, Rognoni M, Cavalieri d'Oro L, Arpaia G, Cimminiello C. Mortality at 30 and 90 days in elderly patients with pulmonary embolism: a retrospective cohort study. Intern Emerg Med. 2015;10:431–6.

37. Baroletti S, Munz K, Sonis J, Fanikos J, Fiumara K, Paterno M, Goldhaber SZ. Electronic alerts for hospitalized high-VTE risk patients not receiving prophylaxis: a cohort study. J Thromb Thrombolysis. 2008;25:146–50.

38. Beeler PE, Kucher N, Blaser J. Sustained impact of electronic alerts on rate of prophylaxis against venous thromboembolism. Thromb Haemost. 2011;106:734–8.

39. Durieux P, Nizard R, Ravaud P, Mounier N, Lepage E. A clinical decision_support system for prevention of venous thromboembolism: effect on physician behavior. JAMA. 2000; 283:2816–21.

40. Fiumara K, Piovella C, Hurwitz S, Piazza G, Niles CM, Fanikos J, Paterno M, Labreche M, Stevens LA, Baroletti S, Goldhaber SZ. Multi-screen electronic alerts to augment venous thromboembolism prophylaxis. Thromb Haemost. 2010;103:312–7.

41. Galanter WL, Thambi M, Rosencranz H, Shah B, Falck S, Lin FJ, Nutescu E, Lambert B. Effects of clinical decision support on venous thromboembolism risk assessment, prophylaxis, and prevention at a university teaching hospital. Am J Health Syst Pharm. 2010;67:1265–73.
42. Haut ER, Lau BD, Kraenzlin FS, Hobson DB, Kraus PS, Carolan HT, Haider AH, Holzmueller CG, Efron DT, Pronovost PJ, Streiff MB. Improved prophylaxis and decreased rates of preventable harm with the use of a mandatory computerized clinical decision support tool for prophylaxis for venous thromboembolism in trauma. Arch Surg. 2012;147:901–7.
43. Janus E, Bassi A, Jackson D, Nandurkar H, Yates M. Thromboprophylaxis use in medical and surgical inpatients and the impact of an electronic risk assessment tool as part of a multi-factorial intervention. A report on behalf of the elVis study investigators. J Thromb Thrombolysis. 2011;32:279–87.
44. Kucher N, Puck M, Blaser J, Bucklar G, Eschmann E, Lüscher TF. Physician compliance with advanced electronic alerts for preventing venous thromboembolism among hospitalized medical patients. J Thromb Haemost. 2009;7:1291–6.
45. Kucher N, Koo S, Quiroz R, Cooper JM, Paterno MD, Soukonnikov B, Goldhaber SZ. Electronic alerts to prevent venous thromboembolism among hospitalized patients. N Engl J Med. 2005;352:969–77.
46. Lecumberri R, Panizo E, Gomez-Guiu A, Varea S, García-Quetglas E, Serrano M, García-Mouriz A, Marqués M, Gómez-Outes A, Páramo JA. Economic impact of an electronic alert system to prevent venous thromboembolism in hospitalized patients. J Thromb Haemost. 2011;9:1108–15.
47. Lecumberri R, Marqués M, Díaz-Navarlaz MT, Panizo E, Toledo J, García-Mouriz A, Páramo JA. Maintained effectiveness of an electronic alert system to prevent venous thromboembolism among hospitalized patients. Thromb Haemost. 2008;100:699–704.

48. Marco P, Lopez-Abadia E, Lucas J. More on thromboprophylaxis: electronic alerts in hospitalized patients at risk of venous thromboembolism. Thromb Haemost. 2008;100:525–6.
49. Mitchell JD, Collen JF, Petteys S, Holley AB. A simple reminder system improves venous thromboembolism prophylaxis rates and reduces thrombotic events for hospitalized patients. J Thromb Haemost. 2012;10:236–43.
50. Piazza G, Rosenbaum EJ, Pendergast W, Jacobson JO, Pendleton RC, McLaren GD, Elliott CG, Stevens SM, Patton WF, Dabbagh O, Paterno MD, Catapane E, Li Z, Goldhaber SZ. Physician alerts to prevent symptomatic venous thromboembolism in hospitalized patients. Circulation. 2009;119:2196–201.
51. Piazza G, Goldhaber SZ. Computerized decision support for the cardiovascular clinician: applications for venous thromboembolism prevention and beyond. Circulation. 2009;120:1133–7.
52. Sobieraj DM. Development and implementation of a program to assess medical patients' need for venous thromboembolism prophylaxis. Am J Health Syst Pharm. 2008;65:1755–60.
53. Streiff MB, Carolan HT, Hobson DB, Kraus PS, Holzmueller CG, Demski R, Lau BD, Biscup-Horn P, Pronovost PJ, Haut ER. Lessons from the Johns Hopkins Multi-Disciplinary Venous Thromboembolism (VTE) Prevention Collaborative. BMJ. 2012;344:e3935.
54. Barbar S, Noventa F, Rossetto V, Ferrari A, Brandolin B, Perlati M, De Bon E, Tormene D, Pagnan A, Prandoni P. A risk assessment model for the identification of hospitalized medical patients at risk for venous thromboembolism: the Padua Prediction Score. J Thromb Haemost. 2010;8:2450–7.
55. Moxey A, Robertson J, Newby D, Hains I, Williamson M, Pearson SA. Computerized clinical decision support for prescribing: provision does not guarantee uptake. J Am Med Inform Assoc. 2010;17:25–33.

56. Moja L, Liberati E, Galuppo L, Gorli M, Maraldi M, Nanni O, Rigon G, Ruggieri P, Ruggiero F, Scaratti G, Vaona A, Kwag K. Barriers and facilitators to the uptake of computerized clinical decision support systems in specialty hospitals: protocol for a qualitative cross-sectional study. Implement Sci. 2014;9:105.
57. Shojania KG, Jennings A, Mayhew A, Ramsay CR, Eccles MP, Grimshaw J. The effects of on-screen, point of care computer reminders on processes and outcomes of care. Cochrane Database Syst Rev 2009, CD001096.
58. Kortteisto T, Raitanen J, Komulainen J, Kunnamo I, Makela M, Rissanen P, Kaila M; EBMeDS (Evidence-Based Medicine electronic Decision Support) Study Group. Patient-specific computer-based decision support in primary healthcare—a randomized trial. Implement Sci. 2014; 9:15.
59. Lachin J, Matts J, Wei L. Randomization in clinical trials: conclusions and recommendations. Control Clin Trials. 1988;9:365–74.
60. Twisk JWR. Applied longitudinal data analysis for epidemiology: a practical guide. 2nd edition. Cambridge: Cambridge University Press; 2013.
61. World Medical Association Declaration of Helsinki - Ethical Principles for Medical Research Involving Human Subjects. http://www.wma.net/en/30publications/10policies/b3/. Accessed April 2016.
62. Taljaard M, Weijer C, Grimshaw JM, Eccles MP; Ottawa Ethics of Cluster Randomised Trials Consensus Group. The Ottawa Statement on the ethical design and conduct of cluster randomised trials: precis for researchers and research ethics committees. BMJ. 2013; 346:f2838.
63. Weijer C, Grimshaw JM, Eccles MP, McRae AD, White A, Brehaut JC, Taljaard M, Ottawa Ethics of Cluster Randomized Trials Consensus Group. The Ottawa Statement on the Ethical Design and Conduct of Cluster Randomized Trials. PLoS Med. 2012;9:e1001346.

64. Hutton JL. Are distinctive ethical principle required for cluster randomized trials? Stat Med. 2001;20:473–88
65. Winkens RA, Knottnerus JA, Kester AD, Grol RP, Pop P. Fitting a routine health-care activity into a randomized trial: an experiment possible without informed consent? J Clin Epidemiol. 1997;50:435–9.
66. Medilogy S.r.l., MediDrug. http://app.medilogy.it/medidrug/. Accessed March 2016.

Table 1. Examples of reminders generated by MediDSS from the EBMeDs and SFINX databases.

Clinical reminders in MediDSS based on EBMeDs database:	*Drug-drug interaction reminders in MediDSS based on SFINX database* [66]
Adjusting warfarin dose in atrial fibrillation:<br>If a patient with atrial fibrillation and warfarin treatment, who has not had heart valve replacement, has not had an INR test during the last 8 weeks, the text “Last INR over 8 weeks ago, order INR?” is shown.<br>If a new INR result is outside the range 1.9–3.2, the text “Check warfarin dose (INR target 2.0–3.0, but note that if the patient has a mechanical mitral valve, the INR target is 2.5–3.5)” is shown, with link to dose calculator.	Interaction between spironolactone and potassium: *“The combination of potassium supplements and potassium sparing diuretics can result in hyperkalemia.”*<br><br>Interaction between warfarin and acetylsalicylic acid: *“Concomitant use is associated with an increased risk of bleeding.”*

Box 1. Description of algorithm for the use of venous thromboembolism prevention therapy

I. The algorithm incorporates the Padua Score [54], which uses ten common risk factors to identify patients at high risk for VTE. Each risk factor is individually weighted according to a point-based scale.

– Active cancer (defined as presence of methastases or recent chemotherapy), known trombophilic condition, and reduced patient mobility are each assigned a score of 3 points.

– Recent major surgery is assigned a score of 2 points.

– Advanced age (greater than 70 years), obesity (BMI greater than 30), bed rest, and hormone replacement therapy or oral contraceptives are each assigned a score of 1 point.

Patients are identified as high-risk for VTE if they accumulate a sum of 4 or more points. When the risk level is low, no medication is recommended; when the risk level is high, a prophylactic strategy using high dosage low-molecular-weight heparin is recommended.

II. The second part of the algorithm involved the Exclusion Criteria for the use of VTE Prophylaxis.

– Home Anticoagulant Therapy

– Contraindications to Pharmacologic Prophylaxis

– Active Bleeding
