# Supplementary material for: Effectiveness of a Hospital-Based Computerized Decision Support System on Clinician Recommendations and Patient Outcomes: A Randomized Clinical Trial
Source: JAMA Netw Open. 2019 Dec 11;2(12):e1917094. doi: 10.1001/jamanetworkopen.2019.17094 (PMC6991299; doi:10.1001/jamanetworkopen.2019.17094)
Supplement: Supplement 2. — Data Sharing Statement [file jamanetwopen-2-e1917094-s002.pdf]

Data Sharing Statement

Moja. Effectiveness of a Hospital-Based Computerized Decision Support System on Clinician Recommendations and Patient Outcomes. *JAMA Netw Open*. Published December 11, 2019. 10.1001/jamanetworkopen.2019.17094

Data
Data available: Yes
Data types: Deidentified participant data
How to access data: Corresponding author: lorenzo.moja@unimi.it
When available: With publication

Supporting Documents
Document types: Statistical/analytic code, Other (please specify)
Additional Information: RCT protocol and statical codes
How to access documents: Corresponding author: lorenzo.moja@unimi.it
When available: With publication

Additional Information
Who can access the data: The investigators will share the trial protocol and statical codes on request (corresponding author at lorenzo.moja@unimi.it).
Types of analyses: Data sharing The investigators will share data (with associated statistical code) used in developing the results presented in this manuscript on request to the corresponding author at lorenzo.moja@unimi.it. Anonymized record level data will be made available on proposal for any analysis by those who have received ethical clearance from their host institution.
Mechanisms of data availability: With investigator support; after having received ethical clearance from ethics committee.
Any additional restrictions: NA
